# Comparative Evaluation of SLA and DLP 3D Printing in Dental Implant Guides: Impact on Fabrication Accuracy, Speed, and Resin Usage

**DOI:** 10.3390/dj13100471

**Published:** 2025-10-16

**Authors:** Michel Beyer, Lena Scheller, Alexandru Victor Burde, Sead Abazi, Adelita Sommacal, Lukas Seifert, Neha Sharma, Florian Markus Thieringer

**Affiliations:** 1 Department of Oral and Cranio-Maxillofacial Surgery and 3D Print Lab, University Hospital Basel, CH-4031 Basel, Switzerland; michel.beyer@usb.ch (M.B.); lena.scheller@unibas.ch (L.S.); sead.abazi@usb.ch (S.A.); adelita.sommacal@usb.ch (A.S.); lukasbenedikt.seifert@usb.ch (L.S.); neha.sharma@usb.ch (N.S.); florian.thieringer@usb.ch (F.M.T.); 2 Medical Additive Manufacturing Research Group (Swiss MAM), Department of Biomedical Engineering, University of Basel, CH-4123 Allschwil, Switzerland; 3 Department of Dental Technology, Faculty of Nursing and Life Sciences, Iuliu Hatieganu University of Medicine and Pharmacy, 400012 Cluj-Napoca, Romania

**Keywords:** 3D printing, stereolithography, dimensional measurement accuracy, dental implants, guided implant surgery

## Abstract

**Background**: Three-dimensional (3D) printing technologies such as Stereolithography (SLA) and Digital Light Processing (DLP) are widely used in dental implantology for the fabrication of surgical guides. While both methods offer clinical viability, their comparative accuracy, efficiency, and material consumption remain subjects of debate. **Objectives:** To compare the dimensional accuracy, printing time, and material consumption of dental surgical guides fabricated using an SLA printer (Formlabs Form 3B) and a DLP printer (NextDent 5100) at various printing orientations. **Methods**: A standardized surgical guide was designed and printed on both printers across seven orientations (0–90°). Five guides per angle were fabricated per technology (n = 35 per printer), scanned, and compared with the CAD reference to evaluate dimensional accuracy. Printing time and resin consumption were recorded. Statistical analyses included the Shapiro–Wilk test and Mann–Whitney U test (α = 0.05). **Results**: Within the evaluated printers and resins, SLA-printed guides demonstrated slightly lower Root Mean Square (RMS) values in most regions, especially in occlusal and drill hole surfaces, while DLP guides tended to undersize Optimal accuracy was observed at 45° for SLA and 60° for DLP. Material consumption was lower for the SLA printer compared with the DLP printer, but SLA required longer printing time (90–200 min vs. 25–75 min for DLP). **Conclusions**: Both technologies produced clinically acceptable guides under the tested conditions. The tested SLA printer tended to offer slightly higher accuracy and material efficiency, whereas the DLP printer achieved shorter printing times, supporting its use in high-throughput workflows. Printing orientation significantly influenced accuracy and resource use.

## 1. Introduction

Three-dimensional (3D) printing, introduced over four decades ago, has evolved rapidly, becoming an essential technology for addressing complex clinical needs across various medical fields [1]. Also referred to as additive manufacturing (AM), this manufacturing technology enables the creation of precise, customized objects directly from digital designs [2]. In the medical field, this capability has led to significant advancements in personalized patient care, especially in developing patient-specific medical devices and surgical planning aids.

In dentistry, 3D printing has brought notable innovations to the conventional manufacturing procedures, enabling precise fabrication of custom dental prosthetics, implants, and surgical guides [3,4]. Unlike the computer-aided subtractive techniques like computer numerical control (CNC) milling, which requires removing material, 3D printing minimizes waste and allows considerable design flexibility, which are essential qualities in clinical environments [5,6,7].

Stereolithography (SLA) and Digital Light Processing (DLP) are the most prominent 3D printing technologies in dentistry [8]. SLA offers high resolution and detail, by using a focused ultraviolet (UV) laser to harden liquid resin in a layer-by-layer fashion, according to the digital design making it suitable for intricate designs. In contrast, DLP enables faster production speeds by curing entire resin layers simultaneously through the use of a DLP projector or using UV light and a digital micromirror device, making it preferable for high-volume applications such as dental aligners and surgical guides [9,10]. Despite their complementary advantages, evidence on whether stereolithography or digital light processing produces more precise guides is conflicting. On one hand, multiple reports suggest that digital light processing may yield more precise guides than stereolithography, such as in the study of Seidel et al., which showed that DLP-printed guides had mean drill-sleeve errors of about 0.09 mm, while SLA guides deviated by roughly 0.29 mm and milled guides by 0.35 mm. Even after steam sterilization all deviations remained within a safe range [11]. Le et al. observed lower coronal and angular deviations with DLP than with SLA in a free-end model [12], and Strunz et al. reported that high-end DLP printers produced implant models with deviations around 0.07 to 0.12 mm compared with 0.26 mm for an SLA printer [13]. On the other hand, Wegmüller et al. found that a consumer DLP printer produced the largest surface deviation (approximately 0.25 mm) and that SLA guides were slightly more accurate [14]. Such conflicting evidence, shaped by differences in printer quality and workflow, underscores the need to examine not only dimensional accuracy but also factors like build orientation, printing time and resin use when comparing these technologies Understanding how these differences in accuracy, speed, and cost-effectiveness influence surgical guide production may therefore help clinicians select the most appropriate technology for a given workflow.

Additionally, for these 3D printing technologies which involve light curing of different types of resins, print orientation is a crucial factor in 3D printing, affecting not only production time and material strength, but also the accuracy of the final model, due to layer alignment and the need for support structures [15,16].

As SLA and DLP technologies gain increasing adoption in clinical dentistry, understanding their performance in terms of both accuracy and efficiency is crucial for guiding clinical decision-making. While accuracy remains a critical factor, other practical considerations, such as material consumption and printing time, also influence the suitability of each technology for routine use [17].

The primary objectives of this study are to:

1. Assess the accuracy of SLA and DLP 3D-printed surgical guides across critical areas, including the mesh surface, occlusal surface, and drill hole surface.
2. Evaluate material consumption and printing time at various printing angles for each technology, aiming to identify efficiency-related factors impacting clinical workflows.

We hypothesized that, under the tested conditions, guides printed with the SLA system (Formlabs Form 3B, Formlabs Inc., Sommerville, MA, USA) would present slightly higher dimensional accuracy and lower resin consumption, whereas guides printed with the DLP system (NextDent 5100, 3D Systems, Rock Hill, SC, USA) would require shorter printing times.

## 2. Materials and Methods

**Figure 1:**
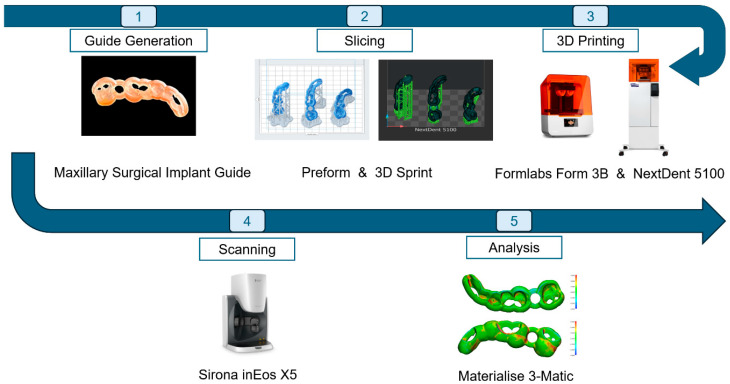
Digital workflow for comparing SLA and DLP 3D printing technologies. The process includes five main steps: (1) surgical guide generation, (2) slicing, (3) 3D printing, (4) scanning, and (5) analysis. Each step is standardized to ensure accuracy and reproducibility.

This study was designed to compare the accuracy of dental implant surgical guides produced using two 3D printing technologies: SLA and DLP. The digital workflow was structured into five main phases: (1) surgical guide generation, (2) slicing, (3) 3D printing, (4) scanning, and (5) analysis. The study workflow is displayed in Figure 1. Each phase was carefully planned to ensure data consistency and reproducibility, enabling a reliable comparison between the two printing methods. As the study involved only in vitro procedures with no patient data, ethical approval was not required.

### 2.1. Data Acquisition

A cone-beam computed tomography (CBCT) scan and a digital intraoral scan of the maxillary arch of a blank typodont (610-200, 3M Unitek, Monrovia, CA, USA) were first obtained to provide accurate anatomical data for virtual planning. Prior to scanning, the crown of tooth 2.6 was completely removed using a rotary instrument to simulate an edentulous site suitable for implant placement. The intraoral scan was performed using a Medit i500 intraoral scanner (Medit Corp., Seoul, Republic of Korea), calibrated according to the manufacturer’s recommendations to ensure optimal precision, while the CBCT scan was performed with Carestream 9300 CBCT unit (Carestream Dental LLC, Atlanta, GA, USA).

Using these scans, a maxillary surgical implant guide with a Straumann T-sleeve (Ø = 5 mm, H = 5 mm; Straumann Holding AG, Basel, Switzerland) was then designed in the dental implant planning software (coDiagnostiX, version 10.4.0; Dental Wings Inc., Chemnitz, Germany). The guide was specifically designed to rest on the teeth in the upper left quadrant, ensuring stability by fitting securely over adjacent teeth. A drilling hole was precisely aligned at the position of tooth 2.6, corresponding to the pre-surgical plan, a wall thickness of 3 mm was generated for the guide which spanned between tooth 2.1 and 2.7 and three seating-check windows were generated proximally of tooth 2.6 and in the anterior region. Once the design was finalized, the guide was exported as a Standard Tessellation Language (STL) file for 3D printing.

### 2.2. Slicing and 3D Printing

#### 2.2.1. Printers and Materials

The STL files, as outlined in Section 2.1, were imported into the slicer software specific for each 3D printer. Slicing parameters and layer thickness were selected, and support structures were generated according to manufacturer’s guidelines. To maintain surface accuracy, the models were oriented with the occlusal surface facing upwards, ensuring that no supports made contact with this surface.

The STL models were oriented at various angles (0°, 15°, 30°, 45°, 60°, 75°, and 90°) to assess the impact of orientation on accuracy, material consumption, and printing time. Five replicas of each guide were printed at each orientation, resulting in a total of 35 models per printer. Details regarding each printer, slicer software, print settings, and materials are summarized in Table 1.

#### 2.2.2. Post-Processing

Upon completion of the printing process, the surgical guides underwent post-processing through a series of standardized steps, according to manufacturer specific protocols (Table 1). Each guide was placed in the washing device filled with isopropyl alcohol (≥99%) to remove excess material. The SLA-printed guides were washed for 20 min (15 min in the Form Wash and 5 min in fresh isopropyl alcohol), while the DLP-printed guides underwent an initial 3 min wash and 2 min rinse in fresh isopropyl alcohol), followed by air drying for at least 30 min.

Subsequently, the guides were cured according to the manufacturer’s instructions: SLA-printed guides were cured for 30 min at 60 °C, while DLP-printed guides were cured for 10 min. After curing, support structures were removed using fine-cutting pliers, and each surgical guide was visually inspected for defects or printing errors.

### 2.3. Digitization of Surgical Guides

The 3D-printed surgical guides were digitized with a Sirona inEos X5 laboratory scanner (Dentsply Sirona, Bensheim, Germany). This scanner was chosen for its reported trueness and precision, coupled with automated calibration and multi-angle stitching, which makes it suitable for evaluating dimensional accuracy in small objects. Before the digitization of each set of guides, the scanner was calibrated by following manufacturer’s instructions and warmed up for optimal accuracy. Each guide was mounted vertically onto the scanning platform, with its distal portion facing the rotation table. To reduce light reflections and enhance surface opacity, the models received a coating of Aesub Yellow (Vertriebs GmbH, Recklinghausen, Germany) self-volatilizing scanning spray applied with an airbrush (nozzle diameter 0.3 mm, 1 bar air pressure) from a distance of approximately 10–15 cm, in a thin and even layer (~2–3 s per surface) to minimize build-up. No direct measurement was performed in this study, but our previous research on similar titanium-dioxide sprays reported mean coating thicknesses of 13–16 µm even when using a in-built conventional pump injector, suggesting that a uniform and very thin layer can be expected under careful application [18].

The five-axis scanner used the automatic jaw-scan mode coupled with multiple HDR exposure to take several multi-angle views for each guide, which were then combined automatically into a single mesh. The scanning process was conducted in a dark room to prevent ambient light interference. 

The resulting meshes were exported as STL files using the inLab software version 18.0 (Dentsply Sirona, Bensheim, Germany). Finally, these STL files of the scanned surgical guides were imported into planning software (Materialise 3-Matic, version 17.0; Materialise NV, Leuven, Belgium) and aligned with the originally designed STL file for accurate registration. The scanned models were aligned to the original CAD design using best-fit surface registration. Support-contact regions were removed to make sure the comparison focused on clinically useful and undistorted areas. Subsequently, deviation analysis was conducted utilizing the 3D deviation comparison tool integrated within Materialise 3-Matic, which calculates point-to-point disparities between surfaces.

### 2.4. Fabrication Accuracy Assessment

The fabrication accuracy of the 3D-printed surgical guides was evaluated by comparing them to the originally planned STL file through three distinct analyses, each targeting critical regions of the surgical guide. First, the Mesh Surface Comparison assessed the entire surface of the surgical guide to evaluate overall accuracy. Second, the Occlusal Surface Comparison, also referred to as seating accuracy, focused on the occlusal surface to verify how well the guide aligned with the teeth, which is essential for the fit and stability of the guide during surgery. Third, the Drilling Hole Surface Comparison examined the inner surface of the drilling hole to ensure precision in implant placement, a critical factor for accurate implant positioning and long-term functionality. These analyses targeted high-precision areas that were unaffected by support structure removal, as both the occlusal and inner drilling hole surfaces were specifically designed to avoid contact with support structures.

For each comparison, the following metrics were calculated: Signed Mean Surface Distance (SMSD), Root Mean Square (RMS), and the Minimal (Min) and Maximal (Max) distances between the 3D-printed and the originally planned surgical guide. The SMSD was used to determine whether the printed models were larger or smaller than the planned mesh, while the RMS provided a measure of absolute differences between the two printing technologies. The formulas for these calculations are displayed in Table 2.

### 2.5. Material Consumption and Printing Time Measurement

To assess the resource efficiency of SLA and DLP printing technologies, material consumption and printing time were measured for each printing angle (0°, 15°, 30°, 45°, 60°, 75°, and 90°) during the production of a single surgical implant guide. Material consumption (in mL) was recorded directly from the printer’s estimated resin usage for each angle, as calculated by the respective slicer software (PreForm version 3.33.0 for SLA and 3D Sprint version 3.1.0.1257 for DLP) while printing time (in minutes) was documented upon print completion. These measurements were collected for both the SLA and the DLP printers, enabling a direct comparison of resource requirements across different printing angles.

### 2.6. Statistical Analysis

Normality of the signed deviation data was assessed using the Shapiro–Wilk test, applied separately for each comparison type (mesh surface, occlusal surface, and drilling hole surface) and for each printing technology (SLA and DLP) across four quantitative metrics: mean, root mean square (RMS), minimum, and maximum deviation. A *p*-value ≥ 0.05 was interpreted as evidence that the data did not significantly deviate from a normal distribution, whereas a *p*-value < 0.05 indicated a statistically significant departure from normality. 

## 3. Results

This study compared the dimensional accuracy and production efficiency of surgical guides fabricated using SLA and DLP 3D printing technologies. Normality of the signed deviation data was tested using the Shapiro–Wilk test, applied separately for each surface region, printing technology (Formlabs- SLA and NextDent- DLP), and metric.

SLA data generally met the assumption of normality for the RMS metric in all regions (Mesh: *p* = 0.18; Occlusal: *p* = 0.22; Cylinder: *p* = 0.15), as well as for mean deviations in the Mesh and Cylinder datasets (*p* = 0.11 and *p* = 0.12, respectively). Minimum and maximum deviations were consistently non-normally distributed (*p* < 0.001). DLP data showed more extensive non-normality, with only RMS in the Mesh and Occlusal datasets approximating normal distributions (*p* = 0.22 and *p* = 0.11, respectively). All other metrics, including mean, minimum and maximum values, showed significant deviation from normality.

Given these results, non-parametric statistical testing was applied. The Mann–Whitney U test was used to compare deviations between SLA and DLP technologies for each metric at each print angulation, based on the non-normal distribution of mean values in the DLP group and inconsistent normality in the SLA group. Each group comparison included *n* = 5 samples per angulation, and statistical significance was defined as *p* < 0.05.

### 3.1. Mesh Surface Comparison

**Figure 2:**
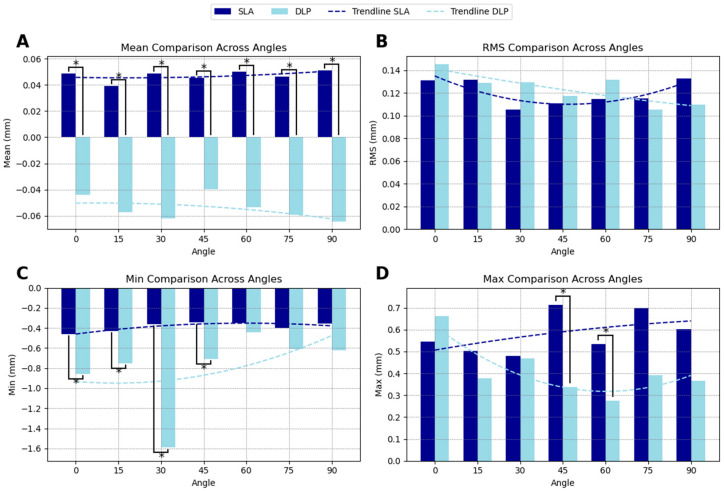
Comparison of mesh surface accuracy for SLA- and DLP-printed surgical guides at different printing angles (0–90°). (**A**) Signed Mean Surface Distance (SMSD) values indicate oversizing (positive) or undersizing (negative). (**B**) Root Mean Square (RMS) deviations show overall accuracy trends. (**C**) Minimum deviation highlights undersizing tendencies in DLP prints. (**D**) Maximum deviation presents oversizing patterns in SLA prints. Asterisks (*) denote statistically significant differences. Trendlines illustrate accuracy trends across angles.

The mesh surface accuracy comparison between SLA and DLP-printed guides demonstrated that SLA-printed guides generally exhibited superior accuracy across most orientations. The SMSD showed a positive deviation for SLA-printed guides (indicating slight oversizing) with an average of 0.047 mm, and a negative deviation for DLP-printed guides (indicating slight undersizing) with an average of −0.054 mm. Statistically significant differences in SMSD were observed at all angles (Figure 2A).

The RMS deviation was generally lower for SLA-printed guides at most orientations, except at 15°, 75°, and 90° printing angles, although the results were not significant. The average RMS deviation amounted to 0.120 mm for SLA and 0.124 mm for DLP. This suggests that SLA technology may provide more consistent accuracy across the mesh surface, as lower RMS values indicate reduced overall deviation from the planned model (Figure 2B).

For the minimum deviation, DLP-printed guides tended to have larger negative deviations compared to SLA-printed guides (−0.797 mm compared to −0.386 mm), shifting the overall deviations toward the negative range. At angles 0°, 15°, 30°, and 45°, the differences between SLA and DLP were statistically significant (Figure 2C).

**Figure A1:**
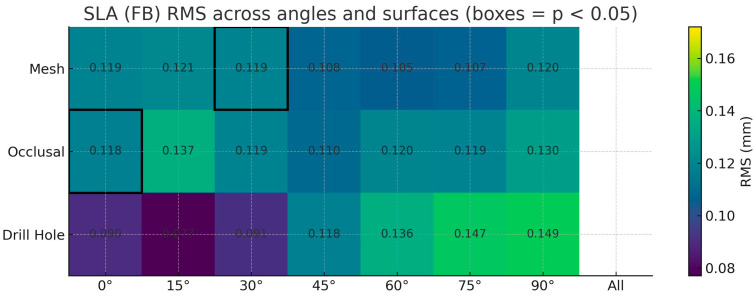
SLA (FB) RMS by surface and printing angle. Boxes mark *p* < 0.05.

**Figure A2:**
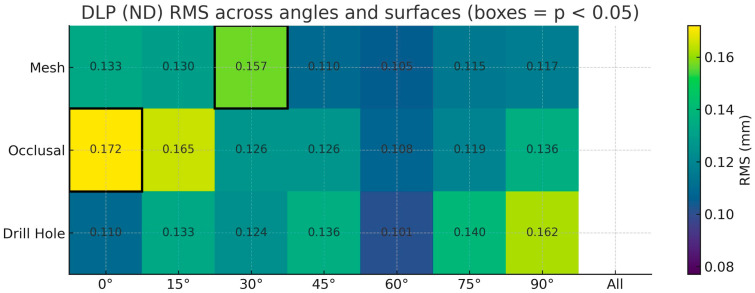
DLP (ND) RMS by surface and printing angle. Color scale identical to Figure A1. Boxes mark *p* < 0.05.

**Figure A3:**
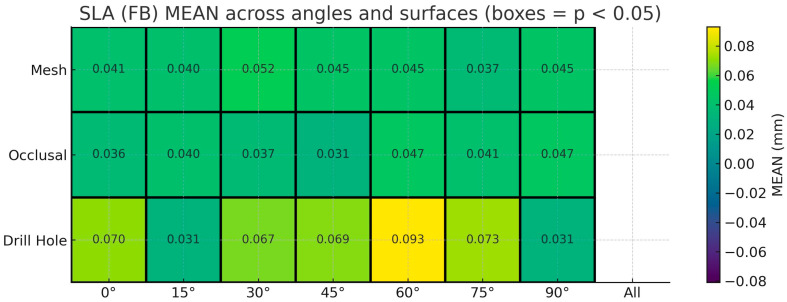
SLA (FB) MEAN by surface and printing angle. Boxes mark *p* < 0.05.

**Figure A4:**
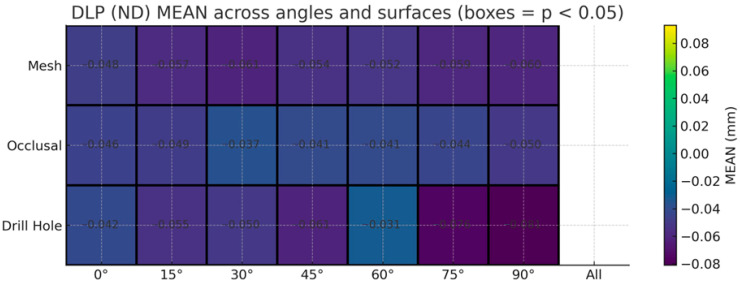
DLP (ND) MEAN by surface and printing angle. Color scale identical to Figure A3. Boxes mark *p* < 0.05.

In terms of maximum deviations, DLP-printed guides exhibited less extreme positive values compared to SLA-printed guides (0.411 mm compared to 0.582 mm). SLA-printed guides displayed significantly larger positive deviations at angles 45° and 60° (Figure 2D). The summarized accuracy outcomes are illustrated in Appendix A (Figure A1, Figure A2, Figure A3 and Figure A4). Detailed numerical results for each printing angle are available in Supplementary Tables S1–S3.

### 3.2. Occlusal Surface Comparison

**Figure 3:**
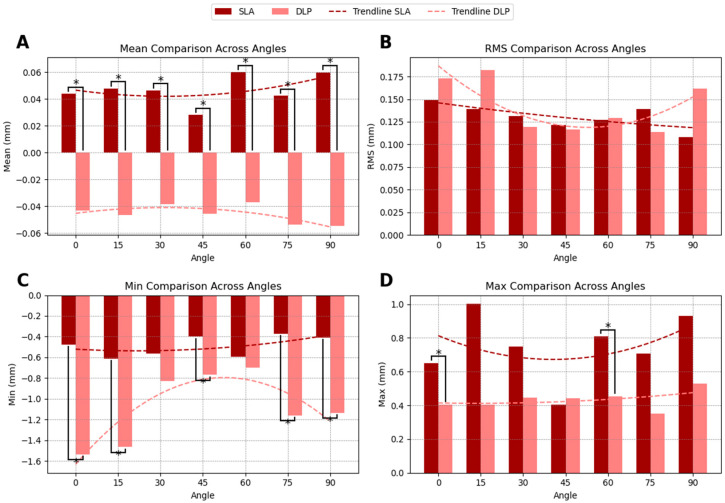
Occlusal surface accuracy of SLA- and DLP-printed surgical guides at different printing angles. (**A**) SMSD values reveal oversizing in SLA guides and undersizing in DLP guides. (**B**) RMS values indicate lower deviations in SLA prints. (**C**) Minimum deviation values show greater dimensional discrepancies in DLP-printed guides. (**D**) Maximum deviations at low angles highlight print orientation effects. Asterisks (*) denote significant differences. Trendlines illustrate accuracy trends across angles.

The occlusal surface accuracy comparison between SLA- and DLP-printed guides revealed distinct trends in dimensional accuracy across printing angles. SLA-printed guides exhibited a slight positive SMSD of 0.047 mm, indicating a tendency for slight oversizing relative to the planned model. In contrast, DLP-printed guides showed a negative SMSD of −0.045 mm, suggesting slight undersizing. Statistically significant differences in SMSD between SLA and DLP were observed at all printing angles (Figure 3A).

RMS deviation was slightly lower for SLA printed guides (0.131 mm) compared to DLP printed guides (0.142 mm), indicating that SLA printing may achieve more consistent accuracy across the occlusal surface, particularly at lower (0°, 15°) and higher (90°) printing angles, even though this difference was not statistically significant (Figure 3B).

In terms of minimum deviation, DLP-printed guides displayed significantly more pronounced negative deviations, with values reaching −1.086 mm, compared to −0.490 mm for SLA-printed guides. This suggests that SLA-printed guides achieve a closer fit to the planned dimensions, with less extreme minimum deviations (Figure 3C).

For maximum deviation, SLA-printed guides exhibited larger positive deviations at certain angles, particularly at 0°, 15°, and 90°, with significant differences observed between SLA and DLP at 0° and 60° (Figure 3D). The summarized accuracy outcomes are illustrated in Appendix A (Figure A1, Figure A2, Figure A3 and Figure A4). Detailed numerical results for each printing angle are available in Supplementary Tables S1–S3.

### 3.3. Drilling Hole Surface Comparison

**Figure 4:**
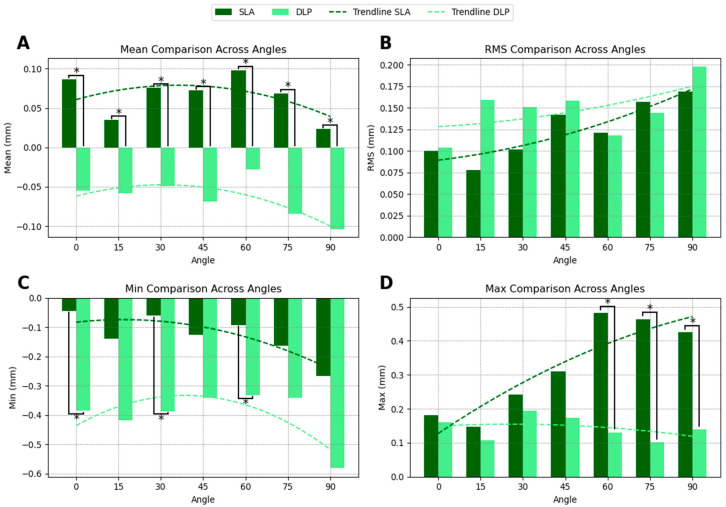
Drilling hole accuracy of SLA- and DLP-printed guides across printing angles. (**A**) SMSD values show DLP-produced guides have slightly smaller drill holes, while S guides exhibit wider holes. (**B**) RMS values indicate overall precision trends. (**C**) Minimum deviations show DLP guides tend to undersize drill holes. (**D**) Maximum deviations reveal greater dimensional variability in SLA guides. Asterisks (*) denote statistically significant differences. Trendlines illustrate accuracy trends across angles.

The comparison of drill hole surface accuracy between SLA- and DLP-printed guides revealed distinct trends across printing angles. SLA-printed guides demonstrated a slight positive SMSD of 0.065 mm, indicating slight oversizing, whereas DLP-printed guides exhibited a negative SMSD of −0.064 mm, suggesting slight undersizing. Statistically significant differences in SMSD were observed at all angles (Figure 4A). However, the variations observed in drill hole size, which are below 0.3 mm, approximate the established tolerance for sleeve fit, indicating adequate path control by both systems.

The RMS deviation was slightly lower for SLA-printed guides, with values of 0.124 mm compared to 0.148 mm for DLP-printed guides, indicating a trend toward more consistent accuracy in drill hole surface dimensions across angles. However, these differences were not statistically significant (Figure 4B).

For minimum deviation, DLP-printed guides showed more pronounced negative values, particularly at angles exceeding 45° (Figure 4C), however these differences were again not statistically significant (Figure 4C).

In terms of maximum deviation, SLA-printed guides displayed larger positive deviations compared to DLP-printed guides, but no statistically significant differences were observed (Figure 4D). The summarized accuracy outcomes are illustrated in Appendix A (Figure A1, Figure A2, Figure A3 and Figure A4). Detailed numerical results for each printing angle are available in Supplementary Tables S1–S3.

### 3.4. Distance Mapping and Orientation Influence

**Figure 5:**
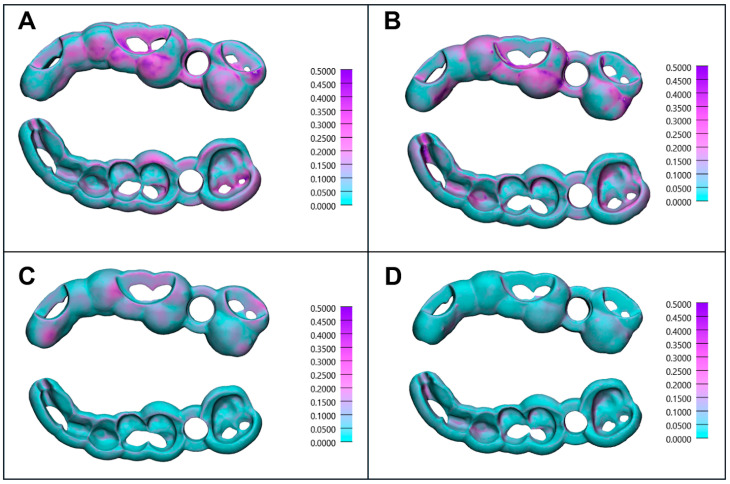
Distance mapping of the scanned surgical implant guides compared to the digitally planned model from the top and bottom views. Scale in millimeters. The worst individual RMS values (Mesh Surface Comparison) for SLA ((**A**)—RMS: 0.193 mm, printing angle: 0°, nr. 4) and DLP-printed models ((**B**)—RMS: 0.192 mm, printing angle: 60°, nr. 1), and the best individual RMS values (Mesh Surface Comparison) for SLA ((**C**)—RMS: 0.074 mm, printing angle: 45°, nr. 5) and DLP-printed models ((**D**)—RMS: 0.065 mm, printing angle: 75°, nr. 4).

Figure 5 presents distance mappings of the scanned surgical implant guides compared to the digitally planned model from top and bottom views, illustrating the impact of printing orientation on accuracy. The worst individual RMS values were observed in SLA-printed guides (Figure 5A—RMS: 0.193 mm, printing angle: 0°, nr. 4) and DLP-printed guides (Figure 5B—RMS: 0.192 mm, printing angle: 60°, nr. 1) highlighting the influence of suboptimal printing angles. Conversely, the best individual RMS values were achieved at a 45° orientation for SLA-printed guides (Figure 5C—RMS: 0.074 mm, printing angle: 45°, nr. 5) and 75° printing angle for DLP-printed guides (Figure 5D—RMS: 0.065 mm, printing angle: 75°, nr. 4).

### 3.5. Material Consumption and Printing Time Comparison

**Figure 6:**
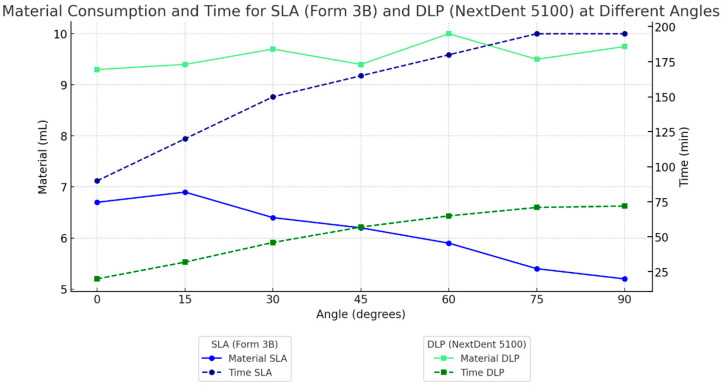
Material consumption and printing time for SLA and DLP-printed surgical guides at various angles. SLA exhibits lower material use, particularly at high angles, while DLP maintains stable material consumption across orientations. (Left axis) Material consumption in mL. (Right axis) Printing time in minutes. SLA printing times increase significantly with angle, while DLP remains more time-efficient.

Material consumption and printing time were evaluated across different printing angles (0°, 15°, 30°, 45°, 60°, 75°, and 90°) for SLA and DLP printers, as shown in Figure 6. For material consumption, SLA showed a notable decrease in resin usage at higher printing angles, reaching a minimum of approximately 5.5 mL at 90°. In contrast, DLP exhibited relatively consistent material consumption across all angles, averaging around 10 mL. This difference indicates that DLP’s material usage is less sensitive to orientation, while SLA material consumption decreases with steeper angles.

In terms of printing time, SLA printing durations increased significantly with angle, ranging from around 100 min at 0° to nearly 200 min at 90°. Conversely, DLP printing times increased only slightly across all angles from 25 min at 0° to 75 min at 90°. These results suggest that DLP is more time-efficient and less affected by print orientation, whereas SLA requires significantly longer print times at higher angles.

## 4. Discussion

### 4.1. Key Findings and Surface Accuracy Comparison

This study compared the dimensional accuracy of SLA (Form 3B) and DLP (NextDent 5100) printing technologies in producing dental implant guides, revealing distinct accuracy patterns. Overall, the tested SLA printing system demonstrated slightly better accuracy, consistent with previous findings by Msallem et al. and Hazeveld et al. [19,20]. Furthermore, in this setup, the results suggest that SLA models tended to be slightly oversized, whereas DLP models were slightly undersized compared to the planned digital design. These dimensional differences may have practical implications for post-processing: the tested SLA system tended to produce slight oversizing, which could allow for easier adjustments, as excess material can be polished off to achieve the desired fit. In contrast, the tested DLP system tended to produce undersizing, which would require pre-print adjustments in CAD software, particularly in high-precision areas such as drilling holes, where adding material post-print is impractical. This observation requires further investigation through additional studies on 3D-printing accuracy to better establish SLA’s advantage in handling intricate features with tight tolerances.

RMS values further support that, in this study, the tested SLA printer tended to show slightly superior precision, as it consistently achieved slightly lower RMS deviations across mesh, occlusal, and drilling hole surfaces. Despite minor variations in accuracy, the tested DLP system demonstrated sufficient precision for general use, making it a practical choice for workflows that prioritize speed over minimal deviations.

In light of these results, several technical elements observed in this study may affect how accurate DLP and SLA guides are manufactured. Seidel et al. demonstrated that DLP-printed guides exhibited lower drill sleeve housing deviations than SLA or milled guides, while varying the layer thickness (50 vs. 100 µm) showed no significant effect [11].

The design and structure of surgical guides also have an impact on the overall accuracy, when a recent systematic review [21] indicated that guides with strong support, such as those attached to teeth or using fixation pins, are more accurate in comparison with guides that rest only on the soft tissue of fully edentulous patients, because soft tissue can be compressed.

Material properties and post-processing steps also influence guide precision. Vara A et al. [22] showed that a biocompatible resin reached roughly 26 µm trueness when printing a full-arch surgical guide, while another reached about 31 µm under the same print settings. This shows that resin makeup and curing methods (like oxygen inhibition compared to nitrogen purge during post-cure) change how polymerization shrinks and affects final sizes.

Finally, sterilization can introduce minor distortions: steam autoclaving has been shown to significantly increase guide deviations, on the order of only a few hundredths of a millimeter, a change that is statistically detectable, but generally considered clinically negligible in guided implant surgery [11].

Several in vitro studies indicate clinically acceptable ranges for dental implant placement and surface deviation of dental implant placement guides. While studies suggest that coronal deviations of 0.5–1.4 mm and apex deviations of 0.76–1.6 mm are clinically acceptable [23] when placing an implant free-hand, for the implants placed by using static surgical guides, there are no well-defined clinically acceptable ranges. However, the manufacturing tolerances of 3D printed dental implant placement guides fall comfortably within the 2 mm safety zone recommended to protect vital anatomical structures [24].

### 4.2. Optimal Printing Angles and Their Impact on Accuracy

This study identified optimal printing angles for accuracy in the tested SLA and DLP systems, with variations depending on surface type.

For the tested SLA printer, a 45° angle yielded the best accuracy for general surfaces (e.g., mesh and occlusal areas), consistent with studies showing that mid-range angles (30–45°) reduce surface deviations and improve dimensional stability [25,26]. For finer and straight features, such as drilling holes, a 15° orientation provided significantly improved accuracy, with an RMS deviation of 0.078 mm. This improvement is likely due to reduced gravitational and layer-stacking distortions at shallower angles [27].

For the tested DLP system, the optimal angle across all surfaces was around 60°, achieving low RMS values (0.124 mm for mesh, 0.142 mm for occlusal surfaces, and 0.148 mm for drilling hole surfaces). This balance between accuracy and structural stability aligns with studies indicating moderate angles in DLP produce stable, accurate prints [28,29]. Accuracy remained consistent at 60° for both general and critical features.

Both tested systems showed increased deviations at extreme angles (0° and 90°), with rising RMS values due to dimensional distortions caused by excessive support requirements and gravitational effects, particularly in the tested SLA printer. Similar findings were reported by Rubayo et al. and Farkas et al. [27,30].

Trendlines confirmed these results: the tested SLA printer’s accuracy decreased as angles increased from 15° to 90°, while the tested DLP system maintained precision up to 60° before deviations increased.

### 4.3. Material Consumption and Printing Time: Efficiency Implications

Material consumption and printing time demonstrated a clear trade-off between the tested SLA and DLP systems. The tested SLA was more material-efficient, particularly at lower printing angles, due to reduced support structure requirements. This efficiency may translate into significant cost savings, especially in high-volume production environments where resin expenses accumulate. In contrast, the tested DLP printer was considerably faster, consistent with studies [31,32] reporting its superior speed in producing clinical guides. This speed advantage makes the tested DLP system more suitable for workflows requiring rapid turnarounds, such as same-day procedures and emergency cases.

In terms of material consumption, the tested SLA system usage ranged from approximately 6.5 mL at a 0° angle to just over 5 mL at 90°, whereas the tested DLP printer consumption remained relatively stable between 9 and 10 mL across angles. For printing time, the tested DLP system significantly outperformed the SLA system, ranging from approximately 25 min at 0° to under 75 min at 90°, compared to SLA’s 90 min to nearly 200 min over the same range. This speed advantage enables potential benefits for high-throughput workflows, such as those in busy dental laboratories or urgent patient cases, enhancing patient care and efficiency, although clinical validation is required. However, this speed advantage comes at the expense of higher resin costs, presenting a key decision point for clinics. The tested SLA system may be better suited for cost-sensitive environments, while the tested DLP printer offers substantial advantages for clinics prioritizing speed and throughput. Balancing these factors is crucial for optimizing operational efficiency and cost-effectiveness in clinical settings.

### 4.4. Cost Analysis Based on Material Consumption and Printing Time

The cost analysis highlights notable differences between the tested SLA and DLP systems in terms of initial and operational expenses. The tested SLA system, represented by the Form 3B, is more affordable, with a setup cost of $7698 [33], compared to the NextDent 5100 DLP printer at $17,954 [34], as detailed in Appendix B. The tested DLP system’s higher cost stems largely from its printer price, making it better suited for fast-paced clinical settings, while the lower cost and material efficiency of the tested SLA system appeals to smaller practices with limited budgets.

SLA’s versatility in handling various resins may help further reduce costs by enabling the use of lower-cost or specialty materials [25]. Conversely, DLP’s faster printing speeds may justify its higher upfront cost for large-scale production environments, despite greater material consumption. Ultimately, the choice depends on clinical priorities. The tested SLA system may be more suitable for smaller practices focusing on cost control, while the tested DLP system is more appropriate for larger settings prioritizing speed and efficiency. These findings align with recommendations emphasizing the importance of matching 3D printing technologies to clinical workflows [25].

### 4.5. Practical Recommendations for Clinical Applications

The findings may provide actionable guidance for clinicians in selecting appropriate 3D printing technologies and settings for surgical guide fabrication:

- General Surgical Guides: For overall accuracy with minimal printing artifacts, mid-range angles (45–60°) appeared optimal in this study for both SLA and DLP, offering stability and precision suitable for general surgical guides.
- SLA for High-Precision Applications: the tested SLA system is more suitable for applications requiring fine detail and precision, particularly for drilling holes or other critical features. Printing at lower angles (15–45°) enhances dimensional stability, providing a cost-effective solution that allows for fine post-processing adjustments if needed. The slight oversizing seen in SLA prints could be corrected via minimal post-processing, offering better control over final fit.
- DLP for Speed-Critical Workflows: the tested DLP printer is recommended for workflows where rapid production is a priority, such as in single-visit procedures or high-throughput workflows. A 60° printing angle yielded balanced accuracy across surfaces, making DLP potentially suitable for splints and general surgical guides requiring quick turnaround times. The minor undersizing observed in DLP prints may even enhance mechanical retention in mucosa-supported guides if the soft tissue compressibility is accounted for.
- All the size differences observed in this study with the tested DLP (undersizing) and SLA systems (oversizing) had a mean surface error of less than 0.3 mm, which is significantly smaller than the 1–2 mm safety margin required to prevent anatomical issues. Knowing about these small differences allows compensation of the designs in the CAD software by utilizing diameter offsets or vertical sleeve placement corrections.

### 4.6. Limitations and Future Directions

Despite promising findings, this study has several limitations. It evaluated only one SLA printer (Form 3B) and one DLP printer (NextDent 5100), along with specific resins, limiting the generalizability of results to other models or materials. The controlled in vitro conditions may not fully replicate clinical variability, and mechanical stress testing was not performed, raising questions about guide durability under surgical conditions.

Standardized support structures and post-processing protocols were used, but variations in support configuration, printing angles, different manufacturer-specific post-processing protocols and clinical practices could impact accuracy. Differences in scanner and software systems, differences in washing and curing conditions as well as resin properties such as shrinkage and thermal expansion, may also influence outcomes. Operator variability in setup and slicing parameters was not assessed but could affect real-world results.

Although our prior studies have quantified spray thickness for comparable products [18], our study did not measure the actual thickness of the applied Aesub layer, which could introduce a minor, uniform offset in deviation analyses.

Another limitation of this study is that material consumption was recorded based on slicer software estimates rather than direct measurement of resin usage. While slicer-generated data provide a convenient and standardized means of comparison, these estimates may not fully reflect actual resin consumption, as they do not account for potential resin losses during printing, post-processing, or support removal.

The application of multiple unadjusted Mann–Whitney U tests to assess variations in surface accuracy metrics across different printing angles represents a statistical constraint in this study. Although this method facilitated a thorough comparison, it elevates the potential for identifying false statistical significance. Considering the limited sample size, the implementation of a multiple comparisons’ correction, such as Bonferroni, would significantly diminish statistical power. Consequently, caution is advised when interpreting *p*-values close to the 0.05 threshold.

In addition, the sample size was limited to five guides per orientation and technology. While this number is in line with other in vitro evaluations, it restricts statistical power and increases the risk of type II errors. No a priori power analysis was performed, which may limit the robustness of conclusions regarding small differences. Future studies with larger sample sizes are therefore warranted to confirm these findings.

Future research should examine a broader range of printers, resins, and clinical conditions while incorporating mechanical stress testing and evaluating diverse post-processing methods to enhance the applicability of these findings.

## 5. Conclusions

This study provides a comprehensive comparison of SLA (Form 3B) and DLP (NextDent 5100) 3D printing technologies for surgical guide fabrication, focusing on accuracy, material efficiency, and clinical applicability. Within the tested printers and conditions, SLA demonstrated slightly better precision in most tests, especially for detailed features such as drilling holes, though differences were not statistically significant. In contrast, DLP offered significantly faster printing times, making it advantageous for time-sensitive workflows, like same-day surgical guides.

Optimal printing angles were identified to enhance accuracy: SLA performed best at 15–45°, while DLP achieved the highest accuracy at a 60° angle. These findings support tailored angle recommendations and emphasize the trade-offs between accuracy, efficiency, and material use, showing that technology and orientation choices can influence workflow outcomes.

Future studies on additional printer models, materials, and clinical conditions are encouraged to further validate these findings.

## Figures and Tables

**Table 1:** Printing and post-processing parameters for SLA (Formlabs 3B) and DLP (NextDent 5100).

Parameter	SLA	DLP
Printer	Formlabs 3B (Formlabs Inc.)	NextDent 5100 (3D Systems)
CAM software	PreForm version 3.33.0	3D Sprint version 3.1.0.1257
Layer thickness	0.1 mm	0.1 mm
Resin	Formlabs Surgical Guide	NextDent SG
Washing station	Form Wash	NextDent Wash
Washing time	15 min	3 min
Rinsing time	5 min	2 min
Curing device	Form Cure	LC-3DPrint Box
Curing time	30 min at 60 °C	10 min

**Table 2:** List of the metrics used in this study with their corresponding formulas and descriptions.

Metric	Formula	Legend
Signed Mean Surface Distance (SMSD)	$\mathrm{Signed}\;\mathrm{MSD}\;=\frac{1}{n_{A}}\left(\sum_{i=1}^{n_{A}}\;\;\underset{p\in P}{min}\;\left[\left\|a_{i}-p\right\|\cdot sign\left(\left(a_{i}-p\right)\cdot n_{p}\right)\right]\right)$	The average signed distance from all points on A (Scanned Object) to their closest points on P (Planning Object).
Root Mean Square (RMS)	$RMS=\sqrt{\frac{1}{n_{A}}\sum_{i=1}^{n_{A}}\;\;{\left(\underset{p\in P}{min}\;\left\|a_{i}-p\right\|\right)}^{2}}$	The square root of the average of the squared signed distances from all points on A (Scanned Object) to their closest points on P (Planning Object).
Minimal (Min)	$d_{\min,\;\mathrm{signed}\;}=\underset{i=1}{\overset{n_{A}}{min}}\;\left(\underset{p\in P}{min}\;\left(\left\|a_{i}-p\right\|\cdot sign\left(\left(a_{i}-p\right)\cdot n_{p}\right)\right)\right)$	The smallest signed distance from any point on A (Scanned Object) to the closest point on P (Planning Object).
Maximal (Max)	$d_{\max,\;\mathrm{signed}\;}=\underset{i=1}{\overset{n_{A}}{max}}\;\left(\underset{p\in P}{min}\;\left(\left\|a_{i}-p\right\|\cdot sign\left(\left(a_{i}-p\right)\cdot n_{p}\right)\right)\right)$	The largest signed distance from any point on A (Scanned Object) to the closest point on P (Planning Object).

## Data Availability

All data supporting the findings of this study are provided in the Supplementary Materials, including Tables S1–S3 (surface comparison values for mesh, occlusal, and drill hole regions for SLA and DLP printers).

## Supplementary Materials

The following supporting information can be downloaded at: https://www.mdpi.com/article/10.3390/dj13100471/s1, Table S1: Mesh surface comparison values (RMS, MEAN, MIN, MAX) for SLA and DLP printers; Table S2: Occlusal surface comparison values (RMS, MEAN, MIN, MAX) for SLA and DLP printers; Table S3: Drill hole surface comparison values (RMS, MEAN, MIN, MAX) for SLA and DLP printers.

## Abbreviations

The following abbreviations are used in this manuscript:

3D	Three-Dimensional
AM	Additive Manufacturing
CAD	Computer-Aided Design
CAM	Computer-Aided Manufacturing
CBCT	Cone-Beam Computed Tomography
DICOM	Digital Imaging and Communications in Medicine
DLP	Digital Light Processing
RMS	Root Mean Square
SMSD	Signed Mean Surface Distance
STL	Standard Tessellation Language
SLA	Stereolithography

## Appendix A

Figure A1 and Figure A2 show the RMS deviations across surfaces and printing angles for SLA and DLP technologies. Darker (cooler) colors indicate smaller deviations (higher precision). Boxes mark cells with statistically significant differences (*p* < 0.05).

Figure A3 and Figure A4 show the MEAN deviations (signed) across surfaces and printing angles. Positive values indicate oversizing; negative values indicate undersizing. Boxes mark cells with statistically significant differences (*p* < 0.05).

## Appendix B

The table below lists the acquisition prices (USD) for the printers, consumables, and essential post-processing equipment used in this study.

**Table A1:** Equipment and material acquisition costs for SLA (Form 3B) and DLP (NextDent 5100) systems.

Price in USD	SLA (Formlabs Inc.)		DLP (Nextdent)	
Printer	Formlabs 3B	$4958	NextDent 5100	$15,904
Resin Tank	Form 3 Resin Tank V2.1	$217	Included	$0
Build Platform	Formlabs Build Platform	$153	Included	$0
Resin (1 L)	Formlabs Surgical Guide	$497	NextDent SG	$364
Washing Station	Form Wash	$764	NextDent Wash	$843
Curing device	Form Cure	$1109	LC-3DPrint Box	$843
TOTAL		$7698		$17,954
