# Supplementary material for: Comparative Evaluation of SLA and DLP 3D Printing in Dental Implant Guides: Impact on Fabrication Accuracy, Speed, and Resin Usage
Source: Dent J (Basel). 2025 Oct 16;13(10):471. doi: 10.3390/dj13100471 (PMC12564608; doi:10.3390/dj13100471)
Supplement: Supplementary file 1 [file dentistry-13-00471-s001.zip › dentistry-3842888-supplementary.pdf]

Supplementary Materials

Table S1. Mesh surface comparison values (RMS, MEAN, MIN, MAX) for SLA and DLP printers.

Angle	Metric	SLA	DLP	*p*-value
0°	MEAN	0.041	-0.048	0.008
	RMS	0.119	0.133	0.095
	MIN	-0.46	-0.771	0.008
	MAX	0.505	0.5	1.0
15°	MEAN	0.04	-0.057	0.008
	RMS	0.121	0.13	1.0
	MIN	-0.377	-0.706	0.008
	MAX	0.534	0.372	0.032
30°	MEAN	0.052	-0.061	0.008
	RMS	0.119	0.157	0.008
	MIN	-0.353	-1.317	0.008
	MAX	0.613	0.416	0.056
45°	MEAN	0.045	-0.054	0.008
	RMS	0.108	0.11	0.841
	MIN	-0.334	-0.648	0.008
	MAX	0.568	0.29	0.008
60°	MEAN	0.045	-0.052	0.008
	RMS	0.105	0.105	1.0
	MIN	-0.36	-0.483	0.151
	MAX	0.536	0.291	0.008
75°	MEAN	0.037	-0.059	0.008
	RMS	0.107	0.115	0.841
	MIN	-0.421	-0.539	0.151
	MAX	0.642	0.338	0.056
90°	MEAN	0.045	-0.06	0.008
	RMS	0.12	0.117	1.0
	MIN	-0.376	-0.572	0.056
	MAX	0.563	0.306	0.032

Table S2. Occlusal surface comparison values (RMS, MEAN, MIN, MAX) for SLA and DLP printers.

Angle	Metric	SLA	DLP	*p*-value
0°	MEAN	0.036	-0.046	0.008
	RMS	0.118	0.172	0.008
	MIN	-0.525	-1.171	0.008
	MAX	0.66	0.384	0.008
15°	MEAN	0.04	-0.049	0.008
	RMS	0.137	0.165	0.056
	MIN	-0.474	-1.207	0.008
	MAX	1.071	0.352	0.008

30°	MEAN	0.037	-0.037	0.008
	RMS	0.119	0.126	0.548
	MIN	-0.512	-0.898	0.151
	MAX	0.67	0.477	0.31
45°	MEAN	0.031	-0.041	0.008
	RMS	0.11	0.126	0.222
	MIN	-0.374	-0.921	0.008
	MAX	0.501	0.373	0.421
60°	MEAN	0.047	-0.041	0.008
	RMS	0.12	0.108	0.222
	MIN	-0.468	-0.678	0.095
	MAX	0.714	0.37	0.008
75°	MEAN	0.041	-0.044	0.008
	RMS	0.119	0.119	1.0
	MIN	-0.382	-1.0	0.016
	MAX	0.732	0.327	0.032
90°	MEAN	0.047	-0.05	0.008
	RMS	0.13	0.136	1.0
	MIN	-0.421	-1.127	0.032
	MAX	0.799	0.444	0.056

Table S3. Drill hole surface comparison values (RMS, MEAN, MIN, MAX) for SLA and DLP printers.

Angle	Metric	SLA	DLP	*p*-value
0°	MEAN	0.07	-0.042	0.008
	RMS	0.09	0.11	0.841
	MIN	-0.046	-0.389	0.008
	MAX	0.17	0.169	0.31
15°	MEAN	0.031	-0.055	0.008
	RMS	0.077	0.133	0.548
	MIN	-0.129	-0.368	0.095
	MAX	0.15	0.127	0.151
30°	MEAN	0.067	-0.05	0.008
	RMS	0.091	0.124	1.0
	MIN	-0.058	-0.324	0.008
	MAX	0.214	0.18	0.151
45°	MEAN	0.069	-0.061	0.008
	RMS	0.118	0.136	0.69
	MIN	-0.115	-0.378	0.032
	MAX	0.295	0.16	0.008
60°	MEAN	0.093	-0.031	0.008
	RMS	0.136	0.101	0.151
	MIN	-0.074	-0.316	0.008
	MAX	0.4	0.156	0.008
75°	MEAN	0.073	-0.076	0.008
	RMS	0.147	0.14	1.0

	MIN	-0.177	-0.435	0.095
	MAX	0.457	0.106	0.008
90°	MEAN	0.031	-0.081	0.032
	RMS	0.149	0.162	0.841
	MIN	-0.251	-0.461	0.222
	MAX	0.401	0.134	0.008
